# Cognition and norms: toward a developmental account of moral agency in social dilemmas

**DOI:** 10.3389/fpsyg.2014.01528

**Published:** 2015-01-07

**Authors:** Leandro F. F. Meyer, Marcelo J. Braga

**Affiliations:** ^1^Institute of Social Environmental and Water Resources, Federal Rural University of the AmazonBelém, Brazil; ^2^Department of Rural Economy, Federal University of ViçosaViçosa, Brazil

**Keywords:** social dilemmas, beliefs, value systems, action logics, developmental psychology, communicative action, institutional analysis

## Abstract

Most recent developments in the study of social dilemmas give an increasing amount of attention to cognition, belief systems, valuations, and language. However, developments in this field operate almost entirely under epistemological assumptions which only recognize the instrumental form of rationality and deny that “value judgments” or “moral questions” have cognitive content. This standpoint erodes the moral aspect of the choice situation and obstructs acknowledgment of the links connecting cognition, inner growth, and moral reasoning, and the significance of such links in reaching cooperative solutions to many social dilemmas. Concurrently, this standpoint places the role of communication and mutual understanding in promoting cooperation in morally relevant conflicts of action in a rather mysterious situation. This paper draws on Habermas’s critique of instrumental action, and on the most recent developments in institutional and behavioral economics with a view to enhancing our knowledge of the interventions used to cope with social dilemmas. We conclude the paper with a brief presentation of a research strategy for examining the capacity of alternative developmental models to predict dissimilar choices under similar incentive conditions in social dilemmas.

## INTRODUCTION

The way one frames an inquiry into any subject has decisive implications. As a quality inherent to the appeal of frameworks, they outline *limited* courses of action and suggest *preferred* approaches to a given subject. These definitions develop from a certain initial consensus of their protagonists, from whence core propositions, assumptions, or premises embody a set of methodological decisions which launch the very conditions of possibility of that research program or discipline. Along with the selected array of observations or data which they disclose, alternative frameworks not only propose diverse guidelines to address a certain problem but also lead to fundamentally distinct viewpoints on the same phenomenon (Kuhn, 1970). Although different perspectives on a complex issue should in principle constitute a better picture, the diverse foundations of alternative theoretical structures and methods of inquiry commonly create barriers to transdisciplinary communication.

The policy implications resulting from inquiries resting on diverse paradigmatic foundations are especially noteworthy when addressing morally relevant conflicts of action such as *social dilemmas*: situations of interdependent choices and outcomes presenting an incongruity between individual and collective gains. Disagreements involving the governance of common-pool resources (CPRs) and the provision of public goods are typical examples. In such situations, assumptions associated with the standard model of “rational actor” and the economic theory of “externalities” present a picture where individuals are viewed as trapped in the “inherent logic” of a situation which “remorselessly generates tragedy” (Hardin, 1968, p. 1244). Regulations imposed by external authorities are then typically recommended in order to make up for the “market failures” and prevent regretful outcomes.

The Bloomington School of Institutional Theory, which received considerable attention from many scholars studying the co-governance of CPRs, has exposed the limits of traditional policy recommendations derived from the conventional economic theory of externalities for addressing the misuse of ecological systems. The institutional analysis and development (IAD) framework, in particular, has helped to better understand why in different circumstances certain social arrangements are sustainable over time while others collapse. Copious field and laboratory research inspired by the IAD framework (e.g., Van Lange et al., 1992; Ostrom et al., 1994; Komorita and Parks, 1995; Lepyard, 1995; Kollock, 1998; Kopelman et al., 2002) has demonstrated that patterns of social interactions and the likelihood of sustainable social arrangements for coping with social dilemmas are affected not only by the structural configuration of the action arena but also by the participants’ personal preferences or valuations regarding the specific content or context of the choices being made.

On the practical side, these developments have enabled policy to move beyond the proposition of universal solutions, implemented by presumably omniscient external authorities, and to recognize the value of the local people’s knowledge basis and community-based management institutions for solving problems involving the appropriation of CPRs and the provision of local public goods in many locations (e.g., Ostrom, 1990, 2003, 2005; Schlager, 1990; McKean, 1992; Hackett et al., 1994; Isaac et al., 1994; Tang, 1994; Baland and Platteau, 1996; Wade, 1996; Agrawal, 2001). On the theoretical side, however, recognition of the most meaningful implications due to Ostrom’s contribution to the field of economic governance requires us to move beyond a simple extension of economic reasoning to address non-market interactions and look to the philosophical foundations of the Bloomington School of Institutional Theory. These foundations, mostly associated with the work of Ostrom (1980, 1982, 1991, 1993), represent an effort to ground institutional theory on a broad vision of the *human condition*, and ultimately suggest that political science has been employing the wrong *paradigm*, and not simply inadequate models or assumptions (Aligica and Boettke, 2009).

Enshrined in the philosophical foundation of the Bloomington School is the view of choice as the fundamental defining element for understanding both human action and social order and change. Without relying on any formal definition of rationality, or reviving the classic “old institutionalist” critique of economics, the Bloomington School holds a sophisticated view on choice: one which attempts to make the principle of methodological individualism coherent with an anthropological and historical understanding of the “human condition and what it is about that condition that disposes human beings to search out arrangements with one another that depend upon organization” (Ostrom, 1982, p. 5).

This perspective significantly broadens the scope of choices under analysis and calls for more flexible assumptions about the actors’ valuations or preferences—-particularly involving intrinsic values and the outcome obtained by others. The Bloomington School suggests that choice in institutional matters refers above all to *choice of ideas, principles and beliefs*, rather than choices about goods and services exchanged in the markets. Comprising the emerging notion of *epistemic choice* (Aligica and Boettke, 2009), the Bloomington School’s perspective (re)introduces into the analysis of rational action challenging matters such as *problem definition*, *representation* of incentives, and *interpretation* of the action situation and environmental feedbacks. Thus, according to the Bloomington School’s view, a theory of institutions and social order should be grounded on an adequate *theory of ideas*—-one that intrinsically links preferences and choices on normative issues to knowledge, learning, and their essential means: language and communication. A comparable emphasis on cognition, learning, and communication also characterizes what North and colleagues propose as the brand new “cognitive institutionalism” (cf. North, 1990, 2005; Denzau and North, 2000; Mantzavinos et al., 2004).

However, it is our contention that the development of the real theoretical, methodological and operational potential associated with the cognitivist approaches to social order has been burdened by a tacit adherence to a specific epistemological conception of rationality and justifiability, known as *foundationalism*, which ultimately denies that “value judgments” or “moral questions” have cognitive content. Since what is not cognitive cannot be rationally justified, this view clearly makes it problematic to develop a theory of rational action aimed at illuminating the role of knowledge, learning processes and communication in coping with morally relevant conflicts of action, like most social dilemmas. As Habermas (1993) observed, admitting that moral judgments *do* have cognitive content, that is, that “they represent more than expressions of contingent emotions, preferences, and decisions of a speaker or actor” (Habermas, 1990, p. 120), is vital for (re)establishing the internal connection between norms and justifying grounds. This connection, which, in Habermas’s terminology, constitutes the *rational foundation of normative validity* (Habermas, 1993, p. 41), is evidently indispensable for admitting that social norms can be justified using arguments, rather than simply imposed by coercion or force. *Moral cognitivism* is likewise needed in order to admit that rational agents can follow norms also on the grounds of their recognition of the norms’ *validity*, rather than on the exclusive basis of utilitarian calculus, or because of some non-rational motive.

This issue pertaining to the epistemology of moral judgments, even though commonly overlooked, is clearly central to the analysis of situations where individual and collective interests conflict; as it is to the Bloomington School’s whole attitude and approach to polycentric order and social dilemmas, viewed as an attempt to give a positive answer to the question posed by Alexander Hamilton in the opening paragraph of *The Federalist* on “whether societies of men are really capable or not of establishing good government from reflection and choice, or whether they are forever destined to depend for their political constitutions on accident and force” (Ostrom, 1971, p. 15). However, though hidden away beneath the IAD framework, the foundationalist epistemology drives its proponents, as most social theorists do, “to assume that instrumental action is the only form of rational action, and that norm-governed action must have some kind of non-rational source” (Heath, 2001, p. 2). It is this epistemological core, in our view, which underlies the Bloomington School’s ultimate adherence to a functionalist explanation of choice on institutional matters (and epistemic choices, more generally), wherein human valuations, ethics, and concern for others are subtly reduced to “a particular form of selection and adaptive behavior,” embodied as “social habits” or “social emotions,” supposedly fixed through gene-culture, co-evolutionary processes (cf. e.g., Boyd and Richerson, 1990, 1992; Bowles and Gintis, 2003; Ostrom, 2005). But then, contrary to what is intended (see cf. Ostrom, 1991, p. 11; Aligica and Boettke, 2009, p. 77), by adding on this functionalist-naturalistic kind of explanation of human sociability, the Bloomington School’s approach to institutions does *not* fundamentally depart from the modes of analysis copied from natural sciences. Actually, what is crucial to explaining the coherence and adaptability of norm-governed systems without abandoning the action frame of reference (and supplying only functionalist explanations of these norms) is the acknowledgment of *moral cognitivism*, which can only be accomplished on the grounds of a dialogical (*non-foundationalist*) conception of rationality and justifiability (cf. Heath, 2008).

As Habermas was one of the first to bring a non-foundationalist epistemology to the task of understanding the logic of social action, his defense of moral cognitivism is integral to his *developmental* account of the human capacity to coordinate interaction through *communicative action*. The cognitivist view on moral judgments is likewise needed for an appreciation of the hypothesis and empirical strategy we put forward in this study, with a view to deepening our knowledge of social dilemmas and supporting the design of more suitable institutions to cope with them.

First, the essentials of the situation in focus are introduced. Without negating the suitability of the usual characterization of the problem in various contexts, we argue against the generalization of the conclusions and policy recommendations which arise from the particular perspective on motivation and rationality assumed in the standard economic approach to the problem. Next, in order to illustrate the inadequacy of such generalizations, we address the disputed role of communication in promoting cooperation among individuals who strive to solve their social dilemmas by themselves. We refer to the framework provided by the Bloomington School of Institutional Theory (Ostrom, 2007; Aligica and Boettke, 2009) and the so-called cognitive institutionalism (Mantzavinos et al., 2004) to show how *epistemic choice*—-and the associated interplay between beliefs, valuations, communication and institutions—-has been addressed within the frames of instrumental action alone. Following Habermas (1984, 1987), we continue to argue that, as this perspective still severs the internal connection between norms and justifying grounds, it conceals the non-instrumental feature of rational action, thus distorting the significance of communication in producing normative agreements in morally relevant conflicts of action. On working as a blind paradigm, foundationalism discredits moral argumentation—-over and above any form of spiritual knowledge—-and redirects both research and teaching about social dilemmas toward an exclusively instrumental-utilitarian approach. While admitting that different methods are performative of different realities (Law and Urry, 2003; Esbjorn-Hargens, 2007), we bring the article to a close by highlighting the significance of moving beyond foundationalism in future education systems in order to restore the rational basis of value judgments and spiritual knowledge, which are believed to be of relevance for better addressing social dilemmas.

## THE ESSENTIALS OF SOCIAL DILEMMAS

So-called social dilemmas include a variety of social interactions in which the individual and collective interests conflict. In a social dilemma, the individual’s sensible self-interested behavior generates a situation in which everyone is worse off. The individuals in question are said to be facing a “social dilemma” because they would all be better off if they found a way to cooperate but there is no incentive for them to bear the costs of cooperation (Ostrom, 2007).

One of the most renowned illustrations of social dilemmas was provided by Hardin (1968) in his much cited *The Tragedy of the Commons*. In that essay, Hardin describes a pasture which is open to all, in which each herdsman receives a private benefit from adding animals to graze on the commons and incurs only delayed costs from his and others’ potential overgrazing. On generalizing the standard model of economic rationality, Hardin goes on to suggest that “as a rational being, each herdsman seeks to maximize his own gain” (ibid, p. 1244). The herdsmen are assumed to pursue short-term, material benefits for themselves and to ignore the immediate consequences for others and the long-term results for all. In Hardin’s text, each rational herdsman concludes that:

…the only sensible course for him to pursue is to add another animal to his herd. And another; and another…. But this is the conclusion reached by each and every rational herdsman sharing a commons. Therein is the tragedy. Each man is locked into a system that compels him to increase his herd without limit—in a world that is limited. Ruin is the destination toward which all men rush, each pursuing his own best interest in a society that believes in the freedom of the commons (Hardin, 1968, pp. 1244–1245).

Many of the most challenging dilemmas, ranging from interpersonal to intergroup issues, are social dilemmas at their core^1^. Furthermore, as Ostrom (2007) remarks, when relatively anonymous individuals independently make decisions which are primarily intended to satisfy their own interests, Hardin’s predictions do tend to be verified, both in the field and in laboratory settings (cf. Ostrom et al., 1994; Cárdenas, 2000; Casari and Plott, 2003; Cárdenas and Ostrom, 2004).

Hardin’s approach may thus be adequate for characterizing a scenario which will lead to the overexploitation of many open access resources, such as pastures, forests, and water sources, and to the pollution of air sheds, landscapes, and water courses. In addressing such problems, the standard theory points to the lack of specific property rights as the only cause of the social dilemmas and does not question the postulates regarding individual motivation and rationality. The policy recommendations which address these problems, therefore, work solely to increase the alternatives which could correct this institutional failure. As Hardin acknowledges, each alternative has its own benefits and drawbacks. “But we must choose,” he says, “or acquiesce in the destruction of the commons (…)” (p. 1245).

In our view, however, the challenge is to show that our inability to cope with many social dilemmas originates less from our belief “in the freedom of the commons,” as Hardin believes, but from our belief in a blinding conception of rationality that compels us to assume that “instrumental action is the only form of rational action, and that norm-governed action must have some kind of non-rational source, such as conditioning, socialization, or habit” (Heath, 2001, p. 2).

This viewpoint erodes the potential of self-organization to solve social dilemmas locally and justifies the intervention of external authorities and the use of coercive means. By assuming that self-interested behavior is the only form of rational behavior, in these circumstances, the standard approach to social dilemmas downplays the moral aspect of the situation and neglects the value of educational practices which are intended to foster responsibility and encourage self-organization. Hardin (1968) himself does so quite directly. For him, in the absence of substantial sanctions, “responsibility is a verbal counterfeit for a substantial *quid pro quo*. It is an attempt to get something for nothing” (p. 1247).

On this basis, Hardin regards any attempt to instill a sense of responsibility in others as “tempting to anyone who wishes to extend his control beyond the legal limits” (p. 1247). He goes so far as to draw on Batson’s “double bind” situation—-hypothesized as being part of the genesis of schizophrenia—-in order to denounce the “serious pathogenic effects” resulting from any appeal to the responsibility of individuals that is intended to regulate social interactions (ibid).

Batson’s double bind theory regarding the origin of schizophrenia involves the same sort of contradiction between verbal and non-verbal communication which Hardin applies in the *Tragedy of the Commons*, thereby implying a necessary skepticism about any plea to “conscience” regarding the regulation of resource use in the absence of sanctions. However, Batson’s double bind principle involves a strong emotional and *unidirectional* message from a mother to her infant, who cannot dispel the anxiety brought about by the contradiction through further communication (cf. Bateson, 1972, 1979; Tarnas, 1999, p. 445). Clearly, linguistic communication among adults working to overcome their collective action problems is different in this respect; what Hardin actually presumes is that moral claims and verbal promises are fundamentally non-binding.

This view regarding the inconsequence of verbal agreements emerges less from a belief in the wicked nature of humans than from a particular epistemological perspective known as *foundationalism*, which eventually leads to *moral non-cognitivism*, i.e., the view that moral ideas have no cognitive content or rational justification. This epistemological underpinning has decisive implications for how we explain rational choice in morally relevant conflicts of action, particularly as it biases our view of the role of communication in producing normative commitments. The following sections expand on the epistemological issue and on how it relates with the developmental perspective on action logics and valuations.

## RECOGNIZING THE EPISTEMOLOGICAL BARRIER TO THE DEVELOPMENTAL PERSPECTIVE ON VALUATIONS

A number of factors have been obstructing recognition of the implications of many reputed developmental models in approaching rational action in social sciences. One basic difficulty ensues from the common belief that psychosocial development refers to childhood and adolescence, that is, to the first 20 years of life. “Traditionally,” as Marchand (2005), puts it, “experts in developmental psychology analyzed the growth of the child and of the adolescent, holding that development ends before adult life begins” (cf. e.g., Inhelder and Piaget, 1955; Piaget, 1970/1972). Were that the case, such a fact would evidently move the developmental framework out of the realm of serious consideration for addressing social dilemmas, since the actors involved in most relevant situations are typically adult humans. Currently, however, researches are coming to an increasing understanding that human subjective and intersubjective developments do indeed have the potential to evolve all the way through adult life (Graves, 1971; Riegel, 1973; Arlin, 1975; Basseches, 1980; Kramer, 1983; Commons and Richards, 1984; Pascual-Leone, 1984; Sinnott, 1984, 1989; Commons et al., 1989; and others).

A second difficulty is that each of the numerous facets or streams of consciousness comprising the overall self appears to have its own internal drives or laws of transformation toward greater complexity and integration. When considered in its entirety, the overall self of particular individuals does not show a sequential or stage-like development, but appears instead as a rather fluid flowing affair, with much overlapping and interweaving (Wilber, 2000, p. 34). The simple intuition of what seems to be an almost infinite number of multiple modalities of individual personalities stirs a natural sense of incommensurability supporting ordinary relativistic objections against the stage developmental framework in general. However, modern psychological structuralism takes all that intertwining into account and entails careful methodological design for assessing particular streams of consciousness and specific self-related competencies, which are defined as capacities not only to solve but also to recognize the very existence of particular types of problems (e.g., empirical-analytic, moral-practical, or interpersonal relationship). Along these lines, as Wilber (ibid) reports, the bulk of research has continued to find that each self-related developmental line itself tends to unfold in a stage-like, sequential, and nested hierarchical fashion, and that self’s center of gravity, so to speak, tends to hover around one basic action logic at any one time (p. 35). Furthermore, according to him, “One of the striking things about the present state of developmental studies is how similar, in broad outline, most of its models are” (p. 5). In fact, by comparing a sizeable number of developmental models and theories, Richards and Commons (1990) also indicate that “[t]he stage sequence [in all of those theories and modes] can be aligned across a common developmental space,” and that, “[t]he harmony of alignment shown suggests a possible reconciliation of [these] theories…” (p. 160; see also Commons, 1981).

Yet, when it comes to the subject of morally relevant conflicts of action, as in social dilemmas, acknowledgment of the developmental framework is obstructed most of all by the common idea that “value judgments” or “moral questions” are rationally undecidable. As Heath (2001) indicates, a critical consequence of this view, often unstated, is that “most social theorists simply assume that any agent who acts on the basis of a moral principle, or social norm, is not rationally justified in doing so” (p. 2). According to him, “This is what underlies the widespread tendency among social theorists to assume that instrumental action is the only form of rational action, and that norm-governed action must have some kind of non-rational source, such as conditioning, socialization, or habit” (ibid). Heath further points to how the presumption of non-rationality tempts one to abandon the action frame of reference and supply purely functionalist explanations for the coherence of norm systems and the adaptability of norm-governed action. This trend is noticeable in the current vogue for the sociobiological evolutionary framework for explaining human sociability and adherence to norms—-visible also in the Bloomington approach to the stability of normative agreements.

As suggested, the previously mentioned obstruction is epistemological in nature. In Heath’s words, the “traditional reason for thinking that normative commitments are irrational, or unjustifiable, depends upon a rather specific conception of rationality and justifiability known as foundationalism” (ibid, p. 2). As Heath (2001) summarizes it, foundationalism is a theory of justification intended to provide an answer to the fundamental problem in epistemology: the problem of infinite regress. Foundationalism suggests that any attempt to justify a given statement inferentially generates an infinite regress of new arguments which could be introduced in support of that statement but which will contain premises which in themselves require justification. The only way to break this cycle, Heath remarks (p. 197), is to use the conclusion as a premise (i.e., use circular reasoning) or simply break the chain of reasons (i.e., make an undefended assumption).

Foundationalism represents an instance of the latter strategy, as it holds that there is a class of “basic” beliefs (also called *foundational* beliefs) which are intrinsically (i.e., non-inferentially) justified by virtue of their empirical content. Foundational beliefs are said to be self-justifying or self-evident to the extent that they are not justified by beliefs or constructs other than sensorial perception.

Because validity claims concerning moral statements and normative commitments cannot be grounded in any direct experience with the physical world, the foundationalist epistemology implies that these judgments are essentially non-cognitive (therein moral non-cognitivism). Even though many social scientists have abandoned positivism as methodological stances, the foundationalist conception of rationality and justifiability is still widely accepted.

This epistemological underpinning has obvious implications for how we explain rational choice in morally relevant conflicts of action. In particular, this view influences our understanding of the role of communication in producing normative commitments in these situations.

While the instrumental conception of rationality does not itself presuppose or depend on any sort of moral non-cognitivism (Heath, 2001), the foundationalist standpoint is probably what explains, for instance, Hardin’s (1968) emphatic repudiation of moral argumentation for addressing the commons dilemmas, as previously illustrated.

## BEYOND FOUNDATIONALISM: TOWARD STAGES OF MORAL REASONING

In the preceding section, we attempted to show that because moral non-cognitivism holds that moral judgments are purely relative, it conflicts with the idea that social norms can be justified using arguments, rather than simply being imposed by coercion or force. It also conflicts with the idea that rational agents can obey norms on the grounds of recognition of the norm’s validity, rather than exclusively considering a utilitarian calculus or some non-rational motive. These limitations motivated Habermas (1984, 1987) to maintain that instrumental models do not provide a sufficient basis for a *general* theory of rational action.

Heath (2001) indicates that a common response to the relativist point of view on morals has been that of accepting the formal component of the foundationalist analysis, and seeking only to deny the narrow empiricist list of belief-types that are claimed to be available for “objective validation.” However, he also notes that these theories suffer from well-known difficulties; thus, the relativist position seems quite strong in this context. Conversely, rooted in the new paradigm of epistemology generated by the linguistic turn, Habermas’s strategy in responding to the relativist stance on morality denies the force of the regress argument entirely and is governed by a non-foundationalist defense of the cognitivist conception of moral judgment.

Following Heath’s (2001) outline, Habermas’s discourse-theoretic view has two basic components. First, Habermas claims that non-cognitivist concerns about the truth-aptness of moral judgments is important only if one assumes that the truth represents some kind of correspondence relationship between sentences and the state of affairs in the world^2^. If one denies that this sort of “objectivity” plays any role in vindicating the truth-claims associated with beliefs, then our ability to justify beliefs has nothing to do with their reference to the physical world (i.e., with the knowledge of what can be enacted using individuals’ ordinary senses and their extension). Similarly, when the relativist questions the ultimate justifiability of moral judgments, the argument is persuasive only if it presupposes a “monological” conception of rational justification (i.e., when justification is tacitly treated as a process which involves only the agent’s cognitive states and the objects of representation). This assumption has the effect of reducing all public practices of justification so that they are either secondary or derivative. However, if one assumes, as Habermas does, that *justification is always dialogical*—a process involving an attempt to justify a claim to some other person, so that justification to others is taken as the primary phenomenon—then there is no *a priori* reason to think that moral questions are any less decidable than empirical or scientific ones.

In summary, Habermas suggests that one can defeat moral non-cognitivism by rejecting the traditional project of analytic epistemology, including both the received (correspondence) theory of truth and the received (foundationalist) view of justification. Heath (2001, p. 198) also suggests that one reason why some theorists have taken this more radical step is that “foundationalism does not offer a very persuasive justification for any kind of belief, including empirical ones.”

Despite the revolutionary tone of this epistemological turn, “the first thing to notice about Habermas’s theory of communicative action” as Heath (2001, p. 13) observes, “is that it is a *typological* theory” (emphasis in the original). In presenting his theory of communicative action, Habermas does not reject the instrumental conception of rationality. As Heath (ibid) explains, Habermas takes as his point of departure that agents have access to a set of different, often incommensurable, standards of choice, or action logics. Communicative action is action governed by a particular standard—-namely, that of achieving *understanding*—-whereas instrumental action is action that is governed by a different standard: that of achieving *success*.

**FIGURE 1:**
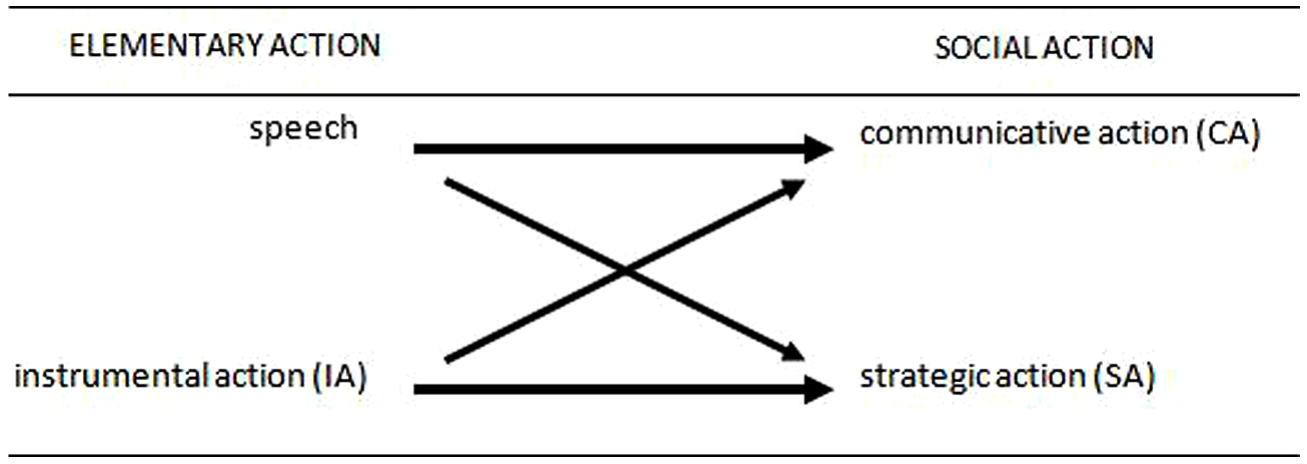
**Elementary action types combine to produce social action types.** *Source:* adapted from Heath (2001, p. 25).

According to Habermas’s typology, *instrumental action* and *speech acts* form two “elementary forms of action” (**Figure 1**). The introduction of a second agent generates social action, understood as a complex phenomenon constructed through interaction between the two elementary forms of action. According to this view, rational agents engaged in social action always face the problem of interdependent expectations, which can be resolved by drawing on the resources of either instrumental action or speech. When the actors are primarily interested in the consequences, social action becomes *strategic action* in the standard game-theoretic sense. However, when speech is used to coordinate the regress of anticipations, it generates that form of action which Habermas characterizes as *communicative action* (cf. Habermas, 1990, p. 133).

This basic scheme is indicated by the straight lines in **Figure 1**. The upward oblique line indicates that communicative action is not the same as speech. Like strategic action, communicative action also presupposes the basic teleological structure of action inasmuch as the actors are assumed to continue to conduct their plans to attain a particular state of affairs. In Habermas’s words, the two social action types differ in that “for the model of strategic action, a structural description of the action directly oriented toward success is sufficient, whereas the model of action oriented toward reaching understanding must specify the propositions of an agreement to be reached communicatively that allows alter to link his action to ego’s” (where the alter and ego are persons; ibid, p. 134). In other words, when engaged in communicative action, actors are assumed to be “prepared to harmonize their plans of action through internal means, committing themselves to pursuing their goals only on the condition of an agreement—-one that already exists or one to be negotiated—-about definitions of the situation and prospective outcomes” (ibid).

Empathy-based justice/fairness concepts are possible key notions in such communicative negotiations. Following the affect-cognition synthesis (cf. Damásio, 1995), contemporary research has pointed to the contribution of empathy to prosocial action, moral judgment, and to resolving caring-justice conflicts (Hoffman, 2000). While viewed as a multidetermined affective response ultimately emerging from natural selection, the biographic arousal of empathy and empathy-charged justice/fairness scripts has been described as a developmental process entrenched within cognition, memory, information processing, and causal attribution (ibid).

When considering the empirical differences in the extent to which different groups or societies depend on explicitly discursive procedures to secure social integration, Habermas offers a plain stage developmental account. In broad lines, his argument is aimed at showing that the stages that occur in his historical reconstruction of the development of communicative action, which takes the form of an interpretation of studies by Durkheim and Mead^3^, are recapitulated in the ontogenesis of our capacity for speech and action, and are isomorphic to the stages described in Laurence Kohlberg’s model of the development of sociocognitive and moral reasoning^4^. The connecting links are provided by Selman (1980) account of sociocognitive development in relation to stages of social perspective taking, which Habermas reformulates in terms of structures of social interaction (see **Table 1**). “The point of this chain of argument is to connect structures of moral judgment to structures of social interaction in such a way that their developmental-logical features stand out more clearly” (McCarthy, 1990, p. ix)^5^.

**Table 1:** Stages of interaction, social perspectives, moral stages, and value systems.

	Cognitive structures						
Types of action (Habermas)	Perspective structure	Concept of authority	Concept of motivation	Social perspective/concept of justice	Stage of moral reasoning (Kohlberg)	Value systems (Graves)
**Preconventional**
Interaction controlled by authority	Reciprocal interlocking of action perspectives (Selman’s Level 2)	Authority of reference persons: externally sanctioning will	Loyalty to reference persons: orientation toward reward and punishments	Egocentric/ complementarity of order and obedience	(1) Punishment and obedience	**3rd** →**4th**
Cooperation based on self-interest				Egocentric/ symmetry of compensation	(2) Naïve instrumental hedonism	**3rd** →**4th**
**Conventional**
Role behavior	Coordination of observer and participant perspectives (Selman’s Level 3)	Internalized authority of supraindividual will: loyalty	Duty *versus* inclination	Primary group perspective/conformity to roles	(3) Good boy good girl morality	** 4th**
Normatively governed interaction		Internalized authority of an impersonal collective will: legitimacy		Perspective of a collectivity / Conformity to the existing systems of norms	(4) Law and order morality	** 4th** →**5th**
**Post-conventional**
Discourse	Integration of speaker and world perspectives (Habermas’s *decentered understanding of the world* orientation)	Ideal *versus* social validity	Autonomy *versus* heteronomy	Principled perspective/orientation toward principles of justice	(5) Morality of democratic contract	**5th**
	
				Procedural perspective/orientation toward procedures for justifying norms	(6) Morality of individual principles	** 6th** →**7th**

*Source: author’s configuration, adapted from Habermas (1990, p. 166) and Graves (2005, p. 443).*

By defining discourse as a *reflective* form of communicative action, Habermas situates the morally relevant presuppositions of practical argumentation as the tail end and point of reference in a constructivist learning process, in which complex forms of social action have given rise to competences resting on repeatedly reorganized sociocognitive inventories and perspective structures which, in turn, have permitted the emergence of more sophisticated forms of action. Viewed within the development of a complex structure of perspectives which culminates in a *decentered understanding of the world,* displayed by subjects who act with an orientation toward reaching understanding, Habermas distinguishes *stages of interaction* in terms of different achievements of coordination, expressing a development that is directed and cumulative.

### COMMUNICATIVE ACTION REQUIRES MORE SOCIOCOGNITIVE CAPACITIES THAN STRATEGIC ACTION

Skipping the details of Habermas’s reconstruction, we single out only those reasons why, according to him, the ability to act from the perspective of a strict concept of morality (as an autonomous and rational sense of duty) can evolve only at the post-conventional level, while the ability for acting strategically requires only an updating of the structure of perspective applying to the preconventional level, without requiring any further reorganization of the sociocognitive inventory.

To show that this occurs, Habermas first redefines the preconventional types of action in terms of *forms of reciprocity* linked to different *structures of behavioral expectations* (not shown in **Table 1**). In this fashion, interaction controlled by authority is redefined in terms of an *asymmetrical form of reciprocity* which tends to operate whenever the authority for controlling the contributions of others to the interaction is unequal, as in the case of the family. Conversely, the *symmetrical form of reciprocity* operates when the participants exercise mutual control over their contributions to the interaction, as for example, in egalitarian friendship (p. 147). These differentiations are correspondingly reflected in two different forms of action coordination: *authority-governed complementarity*, and *interest-governed reciprocity*, to which actors can resort in the face of both cooperative and competitive relationships.

Habermas suggests that authority-governed complementarity and interest-governed symmetrical social relations define *two different types of interaction* which can embody the *same perspective structure*, namely: the reciprocity of action perspectives typical of Selman’s level 2 of perspective taking (**Table 1**). According to Selman, children at this level possess a sociocognitive inventory which enables them to control interactions by deception if necessary. An asymmetry between the developmental requisites for strategic action and action oriented toward reaching understanding starts to emerge as we recognize that in cooperative relationships, the participants *renounce* the use of deception, whereas in authority-governed relationships, the dependent partner *cannot resort* to deception, even in cases of conflict. “Hence, the option of influencing alter’s behavior by means of deception exists only when ego construes the social relationship as symmetrical and interprets the action situation in terms of conflicting needs” (ibid, p. 148).

As shown in **Table 1**, Habermas correlates the justice concept based on the complementarity of order and obedience, which is built into Kohlberg’s first stage of moral reasoning, with the considerations that will guide action when one sees oneself as dependent, and tries to resolve the conflict between ego’s needs and alter’s demands by avoiding threatened sanctions. On the other hand, the concept of justice based on the symmetry of compensation, set in Kohlberg’s second moral stage, emerges only when one starts to see power as distributed equally, and may try to avail oneself of the possibilities for deception which exist in symmetrical relations. Habermas then brings up results from Flavell et al.’s (1968) experiment in order to trace the reorganization of the preconventional stage of interaction and show how strategic action comes to be differentiated from competitive behavior.

In Flavell’s experiment, two cups concealing different amounts of money are put upside down on a table. Each cup bears a label in plain view indicating the payoff value supposedly hidden under the cup. The participants are shown that the relationship between the inscription and the actual amount hidden can be varied at will. Ego’s task is to secretly distribute the payoffs in such a way that alter will fail to guess where the greater amount is hidden. The point of the game is clear: alter will try to win as much as she can, and ego will try to prevent this by means of deception.

Habermas points out that if the participants in the experiment have the perspective structure of Selman’s level 2 (see **Table 1**) they will choose what Flavell called strategy B. Following strategy B, alter chooses the cup labeled “lower payoff,” as she reasons that ego wants to fool her by not concealing the higher payoff under the cup labeled “higher payoff.” On the other hand, participants who are able to engage in Selman’s level 3 of perspective taking will choose Flavell’s strategy C, which is a mixing strategy emerging from the recognition that alter sees through ego’s strategy B. As this mutual (symmetrical) recognition establishes an infinite regress of anticipations, strategy C arises out of alter’s realization that the chance of losing is as great as the chance of winning, no matter what she decides to do.

Habermas suggests that strategy C is characteristic of a type of action only possible at the conventional stage of interaction (**Table 1**), because it requires a coordination of observer and participant perspectives lacking at Selman’s level 2, but necessary for the restructuring of preconventional competitive behavior into strategic action. It is this shift which, according to Habermas, allows ego to attribute stability over time to alter’s pattern of attributes and preferences, so that alter stops being perceived as someone whose actions are determined by shifting needs and interests and begins to be viewed as a subject who intuitively follows rules of rational action. “Beyond this, however, no structural change in the sociocognitive inventory is required. In all other respects the preconventional inventory is adequate for the strategic actor” (1990, p. 150).

On the other hand, as Habermas puts it, the passage to normatively regulated action cannot be adapted so economically to the conventional stage of interaction. According to him, preconventional modes of coordinating action come under pressure in areas of behavior not dominated by competition, where deception is precluded. In these situations the sociocognitive inventory does require a global reconstruction to make room for a mechanism of non-strategic coordination of action. As Habermas explains, this mechanism must be independent of authority relations to an actual reference person and of direct links to self-interests, so that “this stage of conventional but non-strategic action requires basic sociocognitive concepts revolving around the notion of a suprapersonal will” (ibid, p. 152). Habermas then goes on to discuss the structural breaks underlying his justification of the developmental sequence associated with the emergence of different concepts and institutions embodying the idea of a suprapersonal authority, such as loyalty to social roles and legitimacy of rules (see **Table 1**). Concepts and intuitions of this kind provide the elements for constituting a social world of legitimately ordered interpersonal relations and for judging actions according to whether or not they conform to or violate socially recognized norms. At the conventional level, these judgments connect, in turn, with the justice concepts of conformity to roles and conformity to systems of norms, as shown in **Table 1**.

At this point, Habermas indicates that the complex structure of perspectives—-objective, social, and subjective—-underpinning normatively regulated interactions satisfies the structural preconditions of a communicative action in which individual plans of action are coordinated by means of a mechanism for reaching understanding through communication (ibid, p. 158).

According to Habermas, the third stage of interaction, that is, discourse (**Table 1**), takes form only when communicative action becomes fully reflexive. At this stage, the complexity of the perspective structure undertakes further growth in order to make room for the hypothetical attitude which characterizes the decentered understanding of the world and allows participants in argumentation to leave behind the horizon of the unquestioned, intersubjectively shared, non-thematized certitudes of a quasi-natural social world in order to focus on and test validity claims that are initially raised implicitly in communicative action and are naively carried out along with it. As Habermas explains, the structural leap is marked by the synthesis of the two systems of world perspectives and speaker perspectives. “On the one hand, the system of world perspectives, which has been refracted, as it were, by the hypothetical attitude, is [now] constitutive of claims of validity that are thematized in argumentations. On the other hand, the system of fully reversible speaker perspectives is constitutive of the framework within which participants in argumentation can reach rationally motivated agreement” (p. 159).

In discourse, then, the two systems which had been fully developed at the second stage of the conventional level are put in relationship to one another. At the same time, the prior polarity involving communicative and strategic action is overcome in discourse, as the success-orientation of competitors is assimilated into argumentation. As Habermas explains, what happens in argumentation is that “proponents and opponents engage in a competition with arguments in order to convince one another, that is, in order to reach a consensus” (ibid, p. 160). Actually, the condition that arguments are not regressively reduced to mere means of influencing each other, as is often presumed along with an exclusionary instrumental conception of rationality, is what distinguishes the communicative from the strategic use of communication. “In discourse,” Habermas says, “what is called the force of the better argument is wholly unforced” (ibid).

Thus, “[i]n the light of hypothetical claims to validity the world of existing states of affairs is theorized, that is, becomes a matter of theory, and the world of legitimately ordered relations is moralized, that is, becomes a matter of morality” (ibid, p. 161). This “moralization of society” undermines the normative power of the factual, so that institutions which have lost their quasi-natural character can be turned into “so many instances of problematic justice” (ibid). A new reorganization of the fundamental sociocognitive concepts available at the stage of role behavior and normatively governed interaction becomes necessary in order to rationally justify the “uprooted and now free-flowing systems of norms” (ibid). At the post-conventional level, norms of action are subordinated to principles, or higher-order norms. “The notion of the legitimacy of norms of action is now divided into the components of mere de facto recognitions and worthiness to be recognized” (ibid). Correspondingly, a parallel differentiation occurs in the concept of duty, where “respect for the law is no longer considered an ethical motive *per se*” (ibid). To dependence on existing norms is opposed “the demand that the agent make the validity rather than the social currency of a norm the determining ground of his action” (ibid). That is, to autonomy is opposed heteronomy (**Table 1**).

In short, Habermas claims that a strict (cognitivist, universalist, formalist) concept of morality can evolve only at the post-conventional stage, for “only at the post-conventional stage is the social world uncoupled from the stream of cultural givens” (ibid, p. 162). To be sure, it is precisely the sight of plural relativism, which comes into view at the post-conventional stage, which makes the autonomous justification of morality an unavoidable problem (ibid).

Now, if Habermas’s action-theoretic account of the development of the sought-after moral point of view admittedly requires distinctions which are not easy to operationalize, the difficulty in understanding how the conceptions of justice emerge from the sociocognitive inventory of the corresponding stages of interaction can be facilitated by a key insight. This insight, which Habermas properly attributes to Durkheim, is that there is no specific socialization process through which agents acquire moral dispositions. As Habermas puts it, “[i]n trying to understand this process, one has to take into account that the normatively regulated fabric of social relations is moral *in and of itself*, as Durkheim has shown” (ibid, p. 164, emphasis in the original). In Heath’s (2001) words, “This means that acquiring the competences required to manage routine social interactions amounts to acquiring the dispositions and personality structures that we understand to be essential elements of moral agency” (p. 8).

As Habermas plainly recognizes, a hypothetical reconstruction of the type sketched above can at best serve as a guide for further research. With this intent we present, in **Table 1**, Graves’s emergent-stage conception of adult personality systems development. The connecting points were provided by Graves’s own correlation analysis involving the stages advanced in his conception and those described in Kohlberg’s model (cf. Graves, 2005, p. 443)^6^.

Without going on to describe the substance of these correlations, the next section is only aimed at illustrating the implications of our suggested integration of the cognitive-developmental account of moral agency with an analysis of institutional change and development in the context of social dilemmas.

## ON THE PARTIALNESS OF THE BLOOMINGTON SCHOOL’S ACCOUNT OF MORAL CHOICES AND THE ROLE OF COMMUNICATION

**FIGURE 2:**
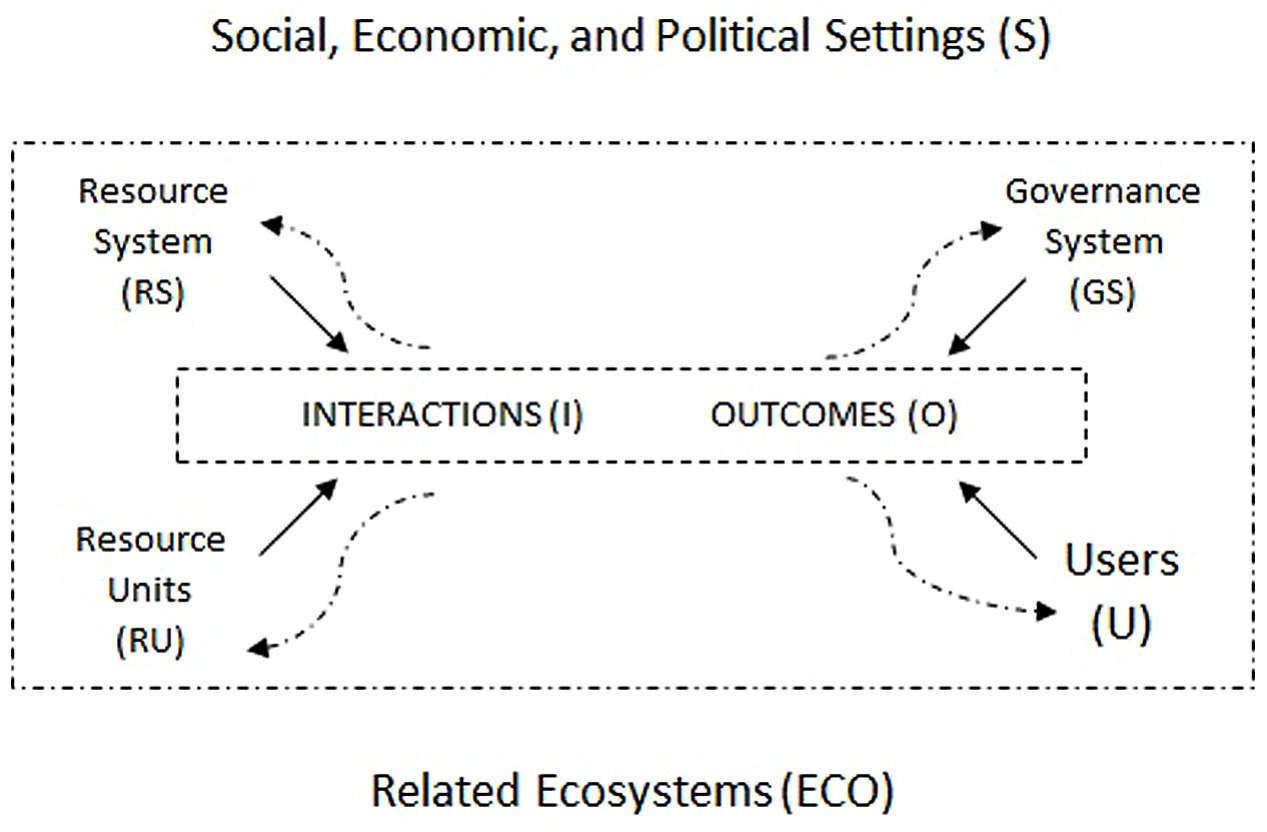
**A multi-tier framework for analyzing interactions and outcomes in a linked social-ecological system (straight arrows represent direct causal links: curved arrows represent feedbacks).** *Source:* Ostrom (2009).

The conceptual scaffoldings supplied by the Bloomington School have undergone a series of transformations since *Governing the Commons* (Ostrom, 1990). **Figure 2** is one of the latest attempts to portray the structure of complex governance problems involving integrated social-ecological systems (SESs). It represents a response to the challenge presented to SES scholars in their search for a proper language “to map and explore the institutional, praxeological, and normative complexity of polycentric systems of human governance” (Aligica and Boettke, 2009, p. 29).

The map starts from the prior recognition that many variables affect the patterns of interactions and outcomes in such systems. It was conceived to help SES scholars “examine the nested attributes of a resource system and the resource units generated by that system that jointly affect the incentives of users within a set of rules crafted by local, distal or nested governance systems to affect interactions and outcomes over time” (Ostrom, 2007, p. 15181).

**Figure 2** shows only the most abstract level of analysis of a generic SES. To investigate real problems in focal SESs, scholars are required to “unpack” each one of the compound units shown in the figure in as many tiers as necessary to reach the relevant data for the particular question under study. When unpacking the component unit “users” (U), Ostrom (2007) draws attention to a series of variables and attributes thought to affect self-organization. The most important variables range from the simple number of users, degree of heterogeneity of the socioeconomic attributes, access to technology and dependence on shared resources, to the different levels of knowledge of the SESs, the possible discrepancy of the mental models of action-outcome linkages, degree to which norms and world views are shared, degree of trust, trustworthiness and reciprocity, and users’ attitudes in relation to leadership and entrepreneurship.

When addressing, in particular, the interplay between cognition, belief systems, valuations and institutions, the Bloomington School assimilates and advances the framework provided by the brand new “cognitive institutionalism,” as termed by Mantzavinos et al. (2004).

Cognitive institutionalism is based on an image of the mind as a complex structure which actively interprets and at the same time classifies the varied signals received by the senses. Updated interpretations and categorizations are configured as a series of “mental models” of the action situation, and are understood as flexible knowledge structures which “gradually evolve during our cognitive development to organize our perceptions and keep track of our memories” (Mantzavinos et al., 2004, p. 76).

**FIGURE 3:**
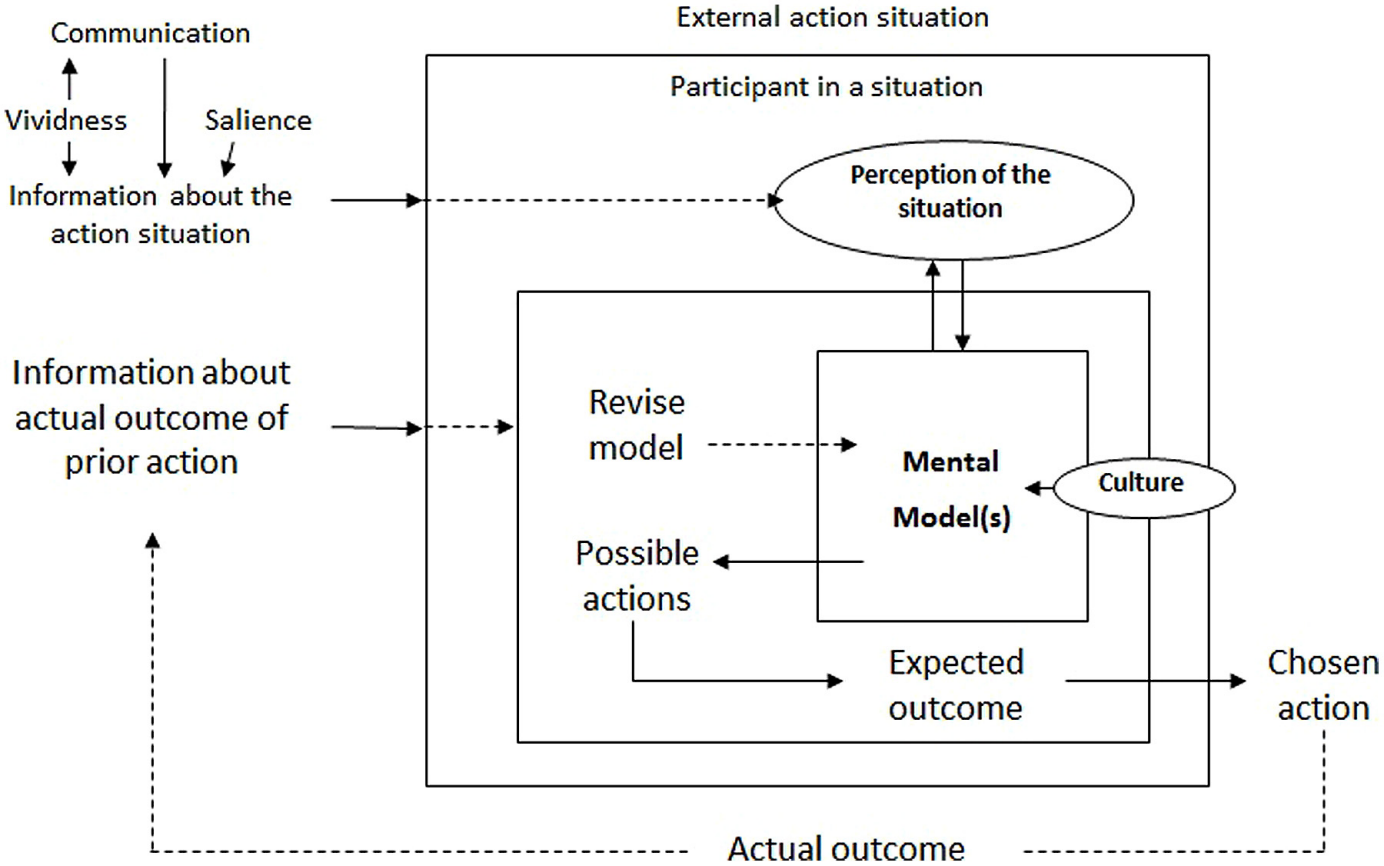
**The impact of communication, vividness, and salience on the relationship between information, action-outcome linkages, and internal mental models.** *Source:* Ostrom (2005).

A pragmatic notion of a mental model, as “the final prediction that the mind makes or expectation that it has regarding the environment before getting feedback from it,” and a behaviorist-like reasoning are adopted to posit the mechanism according to which a given mental model is likely to evolve “according to the feedback received from the environment” (ibid; see **Figure 3**). In this formulation, a “belief” is a “relatively crystallized mental model,” which has become stabilized “when environmental feedback confirms the same mental model many times.” A “belief system” is just the interconnection of beliefs, which can nonetheless be either consistent or inconsistent with such feedbacks. As the authors explain:

Because the mind actively interprets all sensory input, the message regarding the success or failure of the solution attempted will often be misinterpreted. Indeed, the persistence throughout history of dogmas, myths, superstitions, and ideologies based on such flawed belief systems calls us to pay as much attention to learning that produces such beliefs as we do to learning that appears to interpret correctly the problems confronting humans (Mantzavinos et al., 2004, p. 76).

The schema is illustrated in **Figure 3**, which already includes the additions made by Ostrom (2005) to account for two aspects related to the communication opportunities. However, without adding anything substantially different from Hobbes’s original conception of practical rationality, the authors’ interpretation of the truth-value of alternative beliefs hangs clearly on the “old” representationalist theory of cognition and the corresponding monological (foundationalist) model of justification. From this epistemological standpoint, the complex problem related to the likelihood of finding self-organized solutions for collective action dilemmas is just that the individuals involved may come with divergent “mental models” of the action situation, and with divergent and purely subjective normative orientations or preferences on how the dilemma should be solved.

It is at this point that communication emerges as a potentially helpful recourse. However, since the instrumental conception of rationality, underlying the entire analysis, does not support the idea of rational justification of normative principles, communication cannot help in this regard and is inevitably reduced to a vehicle of information exchange: a form of strategic interaction (**Figure 1**) by way of which the agents obtain additional information, based on the experience of others, about likely action-outcome linkages and the feedback they obtain from a situation. Repeated exchange of information could make different mental models converge and thus facilitate coordination (cf. Denzau and North, 2000). When considering the forms and contents of what may be communicated, Frohlich and Oppenheimer (2001) point to the elements of *vividness* and *salience*, which Ostrom (2005), in turn, incorporates into the schema of **Figure 3**.

By taking a position according to which “paying attention is costly,” Frohlich and Oppenheimer explain the importance of these attributes in driving individuals’ attention within the variety of signals they receive.

In order for something to grab one’s attention it must displace something else to which one is attending. To accomplish this, a new focus of attention must have a higher claim. Attention shifts from one object of attention to another as if there were a threshold of value attached to the former which has to be surpassed for the competitor to displace it (Frohlich and Oppenheimer, 2001, p. 8).

While there is nothing wrong with this construction, as far as it goes, it is clearly cramped by the exclusively instrumental account. Since the epistemological underpinning has already ruled out the internal connection between norms and justifying grounds, the “cognitive institutionalisms” cannot really establish a straight link between cognition and institutions. Thus limited, the cognitive capabilities cannot support rational justifications for adopting any governance rule in preference to other feasible alternatives. Correspondingly, the analysis of communication diverts the focus from the essential normative matter toward that of correct interpretations of causal chains.

This limitation, however, is not absolute. Once we have acknowledged Habermas’s epistemological critique, we can distinguish the contribution from psychology’s developmental perspective in interpreting agents’ diverse motivations and styles of reasoning when discussing alternative norms to regulate their interactions in social dilemmas. By selecting appropriated models of adult development, researchers studying social dilemmas can advance testable hypotheses to be falsified in well-established experiments. The next section presents our proposal.

## INTEGRATING DISCOURSE ETHICS AND STAGES OF MORAL AGENCY WITH THE BLOOMINGTON SCHOOL

**FIGURE 4:**
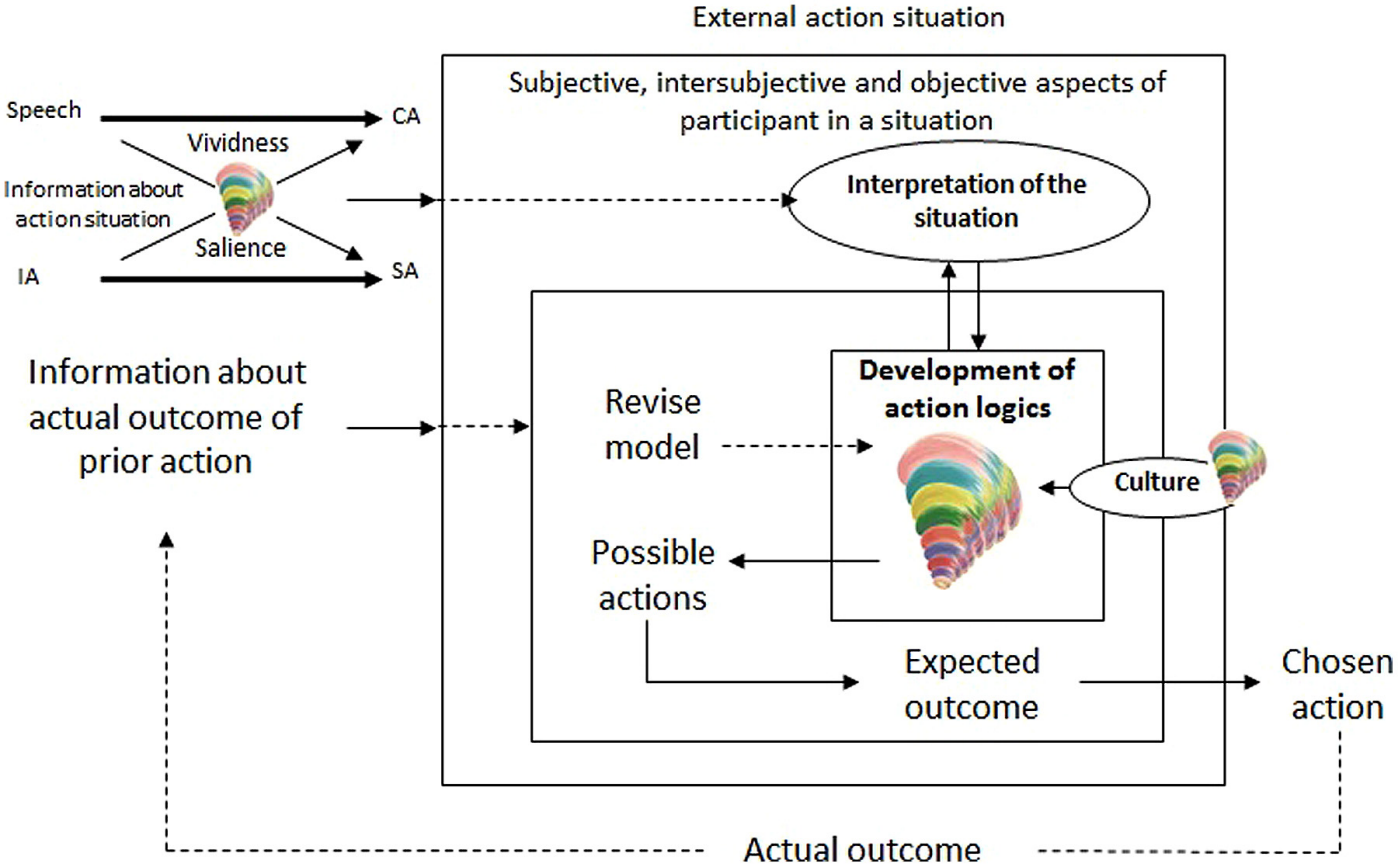
**The impact of the development of action logics and worldviewson individuals’ attitudes toward communication, their perception of the vividness and salience of information, their limitations in revising mental models of action-outcome linkages, and their possible and actually chosen actions.** *Source:* author’s configuration, adapted from Denzau and North (2000) and Ostrom (2005).

The proposed integration can be economically outlined by a simple modification of the previous schema shown in **Figure 3**. In **Figure 4**, we use a pictographic representation of Clare Graves’s (1970) model as the Spiral Dynamics^®^ to symbolize and summarize the entire previous discussion connecting the development of communicative action with the tradition of developmental psychology. In this vein, the small spiral superimposed on Habermas’s typology (in the upper left-hand corner of **Figure 4**) represents Habermas’s own developmental account of the human capacity to coordinate interaction using different standards of choice (**Table 1**), and according to which communicative action emerges only in later (post-conventional) stages of interaction.

According to the summary presented in the previous section, one can reasonably hypothesize that, faced with the conflict of interests that characterizes social dilemmas, individuals centered at the preconventional stages of interaction–third stage in Graves’s model (**Table 1**)—-will use communication as an opportunity to trick others, quite in line with Hardin’s account and the standard game-theoretic prediction. On the other hand, individuals centered at the conventional stages are expected to use communication to reinforce conformity to roles and existing systems of norms, whereas those centered at the discourse stage (post-conventional mode of coordinating action) are expected to use communication as an opportunity to discuss alternative governance rules and to commit themselves to pursue their goals only on the condition of an agreement^7^. The presence of these individuals is probably what explains the regular findings which attest to the effect of communication on enhancing cooperation in both laboratory and field experiments.

In the center of **Figure 4**, the spiral suggests that the internal drives of human development impose restrictions on how individuals can revise their “mental models” of the action situation. On the one hand, centralizations in animistic (second stage), egocentric (third), or authoritarian (fourth) modes of thinking and interacting may explain the “persistence throughout history of dogmas, myths, superstitions, and ideologies” which baﬄes the proponents of cognitive institutionalism. On the other hand, the flexibility which makes it possible for individuals at the discourse stage of interaction to combine the basic attitudes corresponding to the objective, social, and subjective worlds, broadens the interpretation of the “mental models” and the revision process illustrated in **Figure 4**. One can now think not only of revisions anchored in an objectifying attitude faced with the action situation but also of a revision of norm-conformative and expressive attitudes toward it.

The spiral next to the “culture” box stands for both the phylogenetic aspect of Habermas’s historical reconstruction of the development of modes of interaction in human societies and the collective aspect of the ontological recapitulations in the development of individuals. The relevant aspect to discern is that while culture certainly influences and often restricts individual movement up the spiral of interior growth it is *not* its sole determinant. As Wilber (2000) emphasizes, the multiple inner structures configuring human interiority have their own stages of growth and development and are not changed from the outside. The analytical result is that information about cultural values and belief systems are not a substitute for individual information.

In summary, the process through which individuals revise their perceptions of an action situation and then choose an action to seek a particular outcome (including mutual understanding and normative commitments) can allow for the existence of a structured interiority. New limitations and potential to change both perceptions and actions arise from the recognition of the laws of inner transformation. The importance of acknowledging multiple dispositions and forms of rationality in analyzing social dilemmas increases with the recognition that open-ended, multi-stream, complex interior growth is nonetheless a process which involves a continuing *decline in egocentrism*, increasing autonomy and the increasing ability to take other people, places, and things into account when making decisions which affect the well-being of others (cf. Wilber, 2000, 2001).

## CONCLUDING REMARKS: ON FUTURE RESEARCH AND THE ROLE OF EDUCATION

The fact that collective action problems involve fundamentally a moral dilemma is manifest in the opening pages of *The Tragedy of the Commons*, which situate the dilemma within a class of problems whose solution will require a change in human values and ideas of morality. Yet, Hardin’s standpoint on moral judgments is purely relativist. He refers to Fletcher’s (1966) *Situation Ethics* to reveal what he regards as “a not generally recognized principle of morality, namely: the morality of an act is a function of the state of the system at the time it is performed” (p. 1246).

From this perspective, it appears that the role of education is “to reveal to all” the need to abandon belief in the freedom of the commons as long as the “state of the system” requires it. Once this necessity is recognized, “mutual coercion mutually agreed upon” is Hardin’s proposed solution to the problem.

We have no issue with extracting the rationality of a moral choice from the recognition of a contingent necessity. Nevertheless, we believe that the analysis presented here expands the range of alternatives and suggests additional directions for education and research on social dilemmas. Insofar as both communicative rationality and genuine care for others indicate interior dispositions which emerge later on in the path of human development, future research should verify the existence of these traits in social dilemmas using empirical testing. If there is evidence indicating that groups of individuals centered at later stages of interior development are better able to cope successfully with their social dilemmas, then the role of education could include the creation of favorable conditions for humanity to progress up the spiral of interior growth. These conditions would eventually eliminate the need to agree on the necessity of mutual coercion, thus offering a better response to the concern rooted right at the core of the Bloomington School of Institutional Theory.

## Conflict of Interest Statement

The authors declare that the research was conducted in the absence of any commercial or financial relationships that could be construed as a potential conflict of interest.

## Footnotes

1 The famous Prisoner’s Dilemma and the so-called Assurance and Chicken games illustrate key two-person social dilemmas. The provision of public goods and the governance of common-pool resources are typical examples of multiple-person social dilemmas.

2 Habermas is referring to the Correspondence Theory of Truth.

3 This phylogenetic account is in the fifth chapter of The Theory of Communicative Action (1984/1987, v. 2).

4 The ontogenetic ground of Habermas’s onto-phylogenetic parallel is developed in the fourth chapter of Moral Consciousness and Communicative Action (1990). The interested reader may also wish to consult the third chapter of Justification and Application (Habermas, 1993).

5 The authors are aware of and acknowledge the pitfalls of close parallels between phylogenetic and ontogenetic development, like the risk of some kind of teleological progressivism or Euro-America-centrism where “democratic contract” and “individual principles” appear as the highest level to which all developmental processes tend.

6 As previously suggested, we regard contemporary research on the development of empathy as an additional source of indirect validation of Habermas’s account. In Hoffman (2000), for instance, the interested reader will find a comprehensive account of the arousal of empathy, involving a passage from preverbal, automatic, and essentially involuntary modes to higher-order modes of empathy entrenched within the development of cognitive, communicative and perspective-taking capacities.

7 This spiral also reminds us of Graves’s own research on perceptual readiness and validates his point of view (Graves et al., 1965). According to results in this study, the attention mechanism that Frohlich and Oppenheimer (2001) identify as driving the selection of information and perception of the action situation are similarly affected by the dynamics of inner growth; both the vividness and salience of the perceived objects depend on the developmental stage of the observer.
